# Associated factors of sufficient and insufficient physical activity in high school students: a case-control study

**DOI:** 10.3389/fspor.2025.1666033

**Published:** 2025-10-08

**Authors:** Haiwei Li, Haozheng Xu, Jiahao Wang, Yu Chen

**Affiliations:** School of Physical Education, Shanxi Normal University, Taiyuan, China

**Keywords:** physical activity, influencing factors, high school students, case-control study, logistic regression

## Abstract

**Background:**

Insufficient physical activity (PA) among adolescents is a major global public health concern. While theoretical frameworks, such as the Social Ecological Model, highlight multilevel influences—individual, familial, and socio-environmental—on PA behaviors, empirical evidence establishing associations, especially regarding structured PA among high school students in China, remains limited.

**Methods:**

This study used a 1:1 matched case-control design to recruit 222 students from three high schools in Taiyuan City, including 111 participants in the sufficient PA group and 111 matched controls. PA levels in the sufficient group were partly objectively verified using ActiGraph GT3X+ accelerometers. Standardized questionnaires were administered to both groups to assess variables at five levels: individual, interpersonal, organizational, community, and policy. Multivariable logistic regression analyses were performed to identify independent predictors of PA.

**Results:**

At the individual level, self-efficacy was the strongest predictor of PA, with an odds ratio (OR) of 39.45. At the interpersonal level, perceived support from physical education teachers was a significant predictor (OR = 7.96). At the organizational level, adequate access to school PA facilities independently predicted higher engagement in PA (OR = 5.15). Finally, at the community level, multi-channel access to PA information was also a prominent predictor (OR = 11.31).

**Conclusion:**

Self-efficacy, support from physical education teachers, access to adequate school facilities, and multi-channel availability of PA information have been identified as key factors potentially associated with PA engagement among high school students. Therefore, promoting PA in this population may require a comprehensive strategy—one that fosters self-efficacy at the individual level, enhances the support provided by physical education teachers, improves access to and quality of school sports facilities, and strengthens the dissemination of evidence-based PA information through diverse community channels.

## 1 Introduction

Physical activity (PA) is defined as “any bodily movement produced by skeletal muscles that results in energy expenditure” (1) and plays a crucial, evidence-based role in promoting adolescent health. Epidemiological studies show that 81% of adolescents worldwide fail to meet the World Health Organization (WHO) guidelines for aerobic PA (2). Nationally representative surveys from Germany and the United States report that only 20% (3) and 25% (4) of adolescents, respectively, meet these guidelines. Notably, longitudinal evidence shows that declines in PA levels can begin as early as age 7 (5). Meanwhile, the ongoing increase in obesity prevalence, underweight status, and cardiometabolic risks—including type 2 diabetes and stroke—highlights a growing global public health crisis (6, 7).

Unlike early childhood and adulthood, the high school years represent a critical period for establishing lifelong patterns of PA and health-related behaviors. This stage features rapid biological growth and neurocognitive maturation, a period when newly formed habits are more likely to persist into adulthood (8). Developing consistent PA routines and associated positive behaviors during adolescence not only improves academic performance (9), but also establishes a crucial foundation for long-term health and well-being (10). Factors influencing adolescent PA are a critical public health and research concern. This urgency is underscored by Sayyah (11), who found a significant correlation between sports facility access and body composition in school-aged boys.

Previous research has established that changes in adolescent PA are a multifactorial phenomenon that cannot be simply explained (12). The Social Ecological Theory (13) addresses this complexity by providing an integrative framework for examining multilevel influences on PA. At the intrapersonal level, self-efficacy, motivation and attitudes strongly predict PA (14). Interpersonal support from peers, teachers and parents facilitates youth PA (15). Environmental factors—particularly the availability and safety of school and community facilities (16), as well as neighborhood walkability and infrastructure (17)—also play a critical role. And policy-level mandatory PE curricula and municipal investment in public sports facilities significantly influence adolescent PA (18). However, cross-sectional designs (14–18) preclude causal inference and limit generalizability.

To address this methodological gap, the study used a 1:1 matched case-control design, recruiting adolescents who sufficiently participated in structured PA from three public high schools in Taiyuan, Shanxi Province, China. A physically inactive control group was simultaneously selected to allow systematic retrospective comparisons of PA behaviors between the two groups. This design enables the identification of multilevel determinants of sufficient PA while minimizing selection bias and temporal confounding through rigorous matching procedures.

## 2 Methods

### 2.1 Study design and setting

To ensure accurate monitoring of study participants, this study used a 1:1 matched case-control design in Taiyuan Municipality. Cases were defined as high school students who sufficiently engaged in structured PA, while controls were demographically matched peers who did not follow consistent exercise routines.

This study used stratified random sampling to recruit participants from grades 10 to 12 across three high schools in Taiyuan. A total of 1,145 questionnaires were distributed, with 1,066 valid responses, yielding a 93.1% response rate. Based on predefined criteria for sufficient PA, 111 cases meeting the structured exercise threshold were identified. These cases were matched 1:1 with 111 controls based on gender, grade level, and school affiliation, selected from the remaining respondents.

### 2.2 Participants

Based on existing literature (19), We included participants in the sufficient PA group if they reported engaging in sufficient PA for at least 6 months on the Stage of Change Questionnaire, and met the WHO recommendations for adolescent PA, defined by the International Physical Activity Questionnaire—Short Form (IPAQ-SF) as engaging in PA at least 7 times per week for a minimum of 60 min per session. Participants in the control group were in a insufficient PA stage, as assessed by the Stage of Change Questionnaire. In addition, they either did not meet the above PA criteria (assessed by IPAQ-SF). Control participants were matched 1:1 with those in the sufficient PA group by sex, grade, and school.

To reduce self-report bias inherent in questionnaire (20), ActiGraph GT3X + accelerometers (21) were used to objectively verify PA levels in the sufficient PA group. Participants wore the devices positioned at the right anterior axillary line, aligned with the umbilicus. We defined valid data as devices worn for at least eight hours per day on a minimum of three weekdays and one weekend day. After the monitoring period, devices were collected and data were downloaded and analyzed using ActiLife software (version 6.1.4). Before the study began, comprehensive briefings on the research objectives were provided to all participating schools, parents, and students. Written informed consent was obtained from both participants and their legal guardians. The study protocol was approved by the local Institutional Review Board (Approval No. 2025-0504). Demographic and baseline characteristics of participants were summarized in Table 1.

**Table 1:** Basic information of participants.

Variables	Sufficient group (*n* = 111)	Control group (*n* = 111)
Gender (*n*, %)		
Male	93 (83.8)	93 (83.8)
Female	18 (16.2)	18 (16.2)
Grade (*n*, %)		
10th Grade	51 (45.9)	51 (45.9)
11th Grade	36 (32.4)	36 (32.4)
12th Grade	24 (21.6)	24 (21.6)
Age (year)	16.59 ± 0.87	16.60 ± 0.88
Height (cm)	172.44 ± 7.27	172.43 ± 7.24
Weight (kg)	66.71 ± 11.57	66.73 ± 11.52

### 2.3 Study size

Methodological guidelines recommend a minimum sample size of 5–10 observations per predictor variable (22). Based on this criterion, the estimated minimum required sample size for the study was 140 students. To ensure adequate statistical power and accommodate stratified sampling across grade levels, the final analytical sample included 222 students. This represents a 58.6% oversampling above the minimum requirement, improving population representativeness and enhancing the robustness of inferential analyses.

### 2.4 Variables and measures

This study, guided by McLeroy's Social Ecological Model (13), developed a structured questionnaire that covers five hierarchical levels: individual, interpersonal, organizational, community, and policy domains. The instrument used a mixed-methods design, including multiple-choice items, open-ended responses, and 5-point Likert scales (23). The survey gathered demographic information (gender, age, grade, parental education and occupation, household income) and assessed individual-level factors using the Intrinsic Motivation for Physical Exercise Scale (24), revised to better suit 15–18-year-olds and the Self-Efficacy Scale (25), both rated on five-point Likert scales. Interpersonal factors included parental autonomy support, parental exercise habits, and teacher and peer support, primarily assessed through yes/no items and frequency categories. Organizational, community, and policy factors were evaluated through assessments of the school sports environment, natural environmental support, and school policy support, all using yes/no formats. The multidimensional questionnaire, designed according to social ecological theory principles, systematically captures the complex interplay of ecological influences on adolescents' PA behaviors.

Confirmatory factor analysis was conducted to establish the validity of the instrument, focusing on the self-efficacy and intrinsic motivation scales. Two cross-loading items (C13 and C17) were excluded. After their exclusion, principal component analysis with varimax rotation converged after six iterations, extracting three factors based on the eigenvalue >1 criterion. The results showed excellent sampling adequacy (KMO = 0.958) and significant sphericity (Bartlett's *χ*^2^ = 2789.050, df = 66, *p* < 0.001). Test-retest reliability was evaluated after 14 days using Spearman's correlation. As shown in Table 2, the overall scale exhibited strong stability (*r* = 0.868, *p* < 0.01), with subscale coefficients ranging from 0.777 to 0.878—all exceeding the 0.5 threshold—confirming the instrument's robustness.

**Table 2:** Test-retest reliability.

Dimension	Intrinsic motivation	Self-efficacy	Parental autonomy support	Parental exercise habits	Teacher and peer support	School factors at the organizational level	Community level	Policy level	Total
Test-retest reliability	0.859*	0.878*	0.860*	0.818*	0.845*	0.781*	0.868*	0.777*	0.868*

* Indicated that test-retest reliability coefficients were >0.5.

### 2.5 Statistical methodology

Statistical analyses were performed using SPSS version 19.0 (IBM Corp.). Test-retest reliability was evaluated to assess the stability of the measurement instrument. Confirmatory factor analysis was conducted before regression modeling to evaluate construct validity and identify potential multicollinearity among predictor variables (26). Univariate analyses consisted of independent samples *t*-tests for continuous variables and chi-square tests for categorical variables (27). Multivariable binary logistic regression was used to identify independent predictors of PA participation (28). A two-tailed *p*-value of <0.05 was regarded as statistically significant. The interpretation of variable associations and group differences was based on both theoretical frameworks and empirical literature, thereby enhancing the validity of the study's conclusions.

## 3 Results

### 3.1 Characteristics of participants

#### 3.1.1 Accelerometer-measured PA

A subsample of 28 sufficient exercisers (25% of the cohort) underwent 7-day accelerometer monitoring to verify the accuracy of participant screening and confirm established PA patterns. PA levels were categorized based on WHO-recommended MVPA thresholds and Freedson cut-points (29) for intensity classification. Accelerometer data confirmed that all participants met the sufficient exercise criteria (>60 min/day MVPA; Table 3), thereby validating the precision of the screening protocol.

**Table 3:** Accelerometer test results.

Variable	N	Min.	Max.	Mean	S.D.
MVPA (min\weeks)	28	490	1,151	738.14	169.50

#### 3.1.2 Comparative analysis of demographic characteristics between the two groups

Table 4 summarized the baseline characteristics of both groups. The sex distribution was identical across groups (Male: *n* = 93, 83.8%; Female: *n* = 18, 16.2%). In terms of grade composition, the sufficient PA group included 51 students (45.9%) in Grade 10, 36 (32.4%) in Grade 11, and 24 (21.6%) in Grade 12. Economic indicators showed no significant intergroup difference in household monthly income per capita (2,000–6,000 yuan range; *p* > 0.05), but a significant disparity in the distribution of average monthly living expenses (*p* < 0.05). The sufficient PA group predominantly reported 500–1,000 yuan (45.0%), while the control group showed a higher prevalence of ≤500 yuan (43.2%).

**Table 4:** Demographic characteristics of sufficient group and control group.

Title	Category	Sufficient group	Control group	*χ* ^2^	*P*
Average monthly living expenses (*n*, %)	≤500	29 (26.1)	48 (43.2)	8.465	0.037*
	500–1,000	50 (45.0)	35 (31.5)		
	1,000–1,500	22 (19.8)	16 (14.4)		
	≥1,500	10 (9.0)	12 (10.8)		
Monthly per capita household income (*n*, %)	500–700	0 (0.0)	5 (4.5)	9.83	0.08
	700–1,000	2 (1.8)	3 (2.7)		
	1,000–2,000	10 (9.0)	18 (16.2)		
Father's Education Level	Elementary School or Below	11 (9.9)	11 (9.9)	4.287	0.369
	Junior High School	31 (27.9)	37 (33.3)		
	High School or Vocational School	30 (2**7**.0)	30 (27.0)		
	College Diploma	26 (23.4)	15 (13.5)		
	Bachelor's Degree or Above	13 (11.7)	18 (16.2)		
Mother's Education Level	Elementary School or Below	6 (5.4)	15 (13.5)	4.483	0.304
	Junior High School	39 (35.1)	38 (34.2)		
	High School or Vocational School	33 (29.7)	26 (23.4)		
	College Diploma	13 (11.7)	14 (12.6)		
	Bachelor's Degree or Above	20 (18.0)	18 (16.2)		
Father's Occupation	Temporary Worker, Unemployed, or Job Seeking	11 (9.9)	15 (13.5)	1.529	0.981
	Manual Laborer or Self-Employed	33 (29.7)	33 (29.7)		
	Production or Transport Equipment Operator	13 (11.7)	11 (9.9)		
	Agricultural, Forestry, Animal Husbandry, Fishery, and Water Conservancy Worker	12 (10.8)	13 (11.7)		
	Commercial or Service Industry Worker	12 (10.8)	13 (11.7)		
	Civil Servant or Company Employee	9 (8.1)	9 (8.1)		
	Professional Technical Personnel	7 (6.3)	7 (6.3)		
	Government, Public Institution, or Corporate Manager	14 (12.6)	10 (9.0)		
Mother's Occupation	Temporary Worker, Unemployed, or Job Seeking	17 (15.3)	26 (23.4)	9.248	0.235
	Manual Laborer or Self-Employed	24 (21.6)	27 (24.3)		
	Production or Transport Equipment Operator	3 (2.7)	6 (5.4)		
	Agricultural, Forestry, Animal Husbandry, Fishery, and Water Conservancy Worker	10 (9.0)	9 (8.1)		
	Commercial or Service Industry Worker	20 (18.0)	13 (11.7)		
	Civil Servant or Company Employee	12 (10.8)	16 (14.4)		
	Professional Technical Personnel	10 (9.0)	8 (7.2)		
	Government, Public Institution, or Corporate Manager	15 (13.5)	6 (5.4)		

* *p* < 0.05.

### 3.2 Univariate logistic regression analysis of influencing factors

The results of the univariate analysis were summarized in Table 5. Significant differences were found between the sufficient exercise group and the control group for the following variables: intrinsic motivation, self-efficacy, parental support knowledge, parental emotional support, parental investment, agreement with parents' views, openness of parental discussion, parents' exercise duration, support from PE teachers, encouragement and companionship from peers, availability of school sports facilities, provision of extra exercise time at school, availability of school sports competitions, participation in school track meets, adequacy of community sports facilities and venues, community-organized sports events, participation in community sports activities, free skills training, access to other sports knowledge, standards of school physical education curricula, the impact of PE grades, and the physical fitness tests (all *p* < 0.05).

**Table 5:** Univariate analysis.

Factor	Sufficient group (*n* = 111)	Control group (*n* = 111)	t	χ^2^	*p*
Individual level					
Int. Motiv.	3.94 ± 0.92	2.88 ± 1.02	−8.134	–	0.000**
Self-effic.	3.58 ± 0.82	2.35 ± 0.83	−11.185	–	0.000**
Interpersonal Level Social Support					
Par. Supp. Knowl. (D1)	Can	81 (73.0)	47 (42.3)	21.329	0.000**
	Cannot	30 (27.0)	64 (57.7)		
Par. Emot. Supp. (D2)	Can	94 (84.5)	55 (49.5)	31.004	0.000**
	Cannot	17 (15.3)	56 (50.5)		
Par. Invest. (D3)	Can	90 (81.1)	50 (45.0)	30.941	0.000**
	Cannot	21 (18.9)	61 (55.0)		
Agree Views (D4)	Can	97 (87.4)	54 (48.6)	38.287	0.000**
	Cannot	14 (12.6)	57 (51.4)		
Par. Openness (D5)	Can	92 (82.9)	67 (60.4)	13.851	0.000**
	Cannot	19 (17.1)	44 (39.6)		
Par. Exer. Freq. (D6)	≤2 times/wks	56 (50.5)	70 (63.1)	3.647	0.302
	3–4 times/wks	35 (31.5)	27 (24.3)		
	5–6 times/wks	10 (9.0)	7 (6.3)		
	≥6 times/wks	10 (9.0)	7 (6.3)		
Par. Exer. Dur. (D7)	≤2 months	61 (55.0)	80 (72.1)	9.795	0.020*
	3–4 months	29 (26.1)	23 (20.7)		
	5–6 months	9 (8.1)	5 (4.5)		
	≥6 months	12 (10.8)	3 (2.7)		
Par. Exer. Time (D8)	≤30 min	54 (48.6)	62 (55.9)	3.804	0.149
	30–60 min	40 (36.0)	41 (36.9)		
	≥60 min	17 (15.3)	8 (7.2)		
PE Teacher Supp. (D9)	≤1 time/mos	22 (19.8)	68 (61.3)	42.858	0.000**
	2–5 times/mos	53 (47.7)	33 (29.7)		
	≥5 times/mos	36 (32.4)	10 (9.0)		
Peer Encour. (D10)	≤1 time/mos	14 (12.6)	50 (45.0)	30.503	0.000**
	2–5 times/mos	44 (39.6)	35 (31.5)		
	≥5 times/mos	53 (47.7)	26 (23.4)		
Peer Partic. (D11)	No	26 (23.4)	86 (77.5)	64.87	0.000**
	Yes	85 (76.6)	25 (22.5)		
Organizational Level					
Sch. Sports Fac. (E1)	No	25 (22.5)	56 (50.5)	18.68	0.000**
	Yes	86 (77.5)	55 (49.5)		
Extra Exer. Time (E3)	No	46 (41.4)	70 (63.1)	10.399	0.001**
	Yes	65 (58.6)	41 (36.9)		
Sch. Sports Comp. (E4)	No	68 (61.3)	98 (88.3)	29.781	0.000**
	Yes	43 (38.7)	13 (11.7)		
Sch. Track Meet (E5)	No	60 (54.1)	97 (87.4)	9.969	0.020*
	Yes	51 (45.9)	14 (12.6)		
Community Level					
Comm. Sports Fac. (F1)	No	20 (18.0)	41 (36.9)	9.969	0.002**
	Yes	91 (82.0)	70 (63.1)		
Comm. Sports Fac. Adeq. (F2)	No	43 (38.7)	71 (64.0)	14.136	0.000**
	Yes	68 (61.3)	40 (36.0)		
Comm. Sports Events (F3)	No	49 (44.1)	84 (75.7)	22.975	0.000**
	Yes	62 (55.9)	27 (24.3)		
Comm. Sports Partic. (F4)	No	55 (49.5)	97 (87.4)	36.805	0.000**
	Yes	56 (50.5)	14 (12.6)		
Free Train. (F5)	No	55 (49.5)	97 (87.4)	36.805	0.000**
	Yes	56 (50.5)	14 (12.6)		
Other Knowl. (F6)	No	22 (19.8)	76 (68.5)	53.271	0.000**
	Yes	89 (80.2)	35 (31.5)		
Policy Level					
Sch. PE Assess. (G1)	No	22 (19.8)	52 (46.8)	18.243	0.000**
	Yes	89 (80.2)	59 (53.2)		
Phys. Fit. Test (G2)	No	26 (23.4)	56 (50.5)	17.404	0.000**
	Yes	85 (76.6)	55 (49.5)		
PE Grades Impact (G3)	No	44 (39.6)	44 (39.6)	0	1
	Yes	67 (60.4)	67 (60.4)		

* *p* < 0.05.

** *p* < 0.01.

### 3.3 Multivariate logistic regression analysis of influencing factors

This study initially conducted univariate regression to screen variables and assessed multicollinearity. All 23 variables had variance inflation factor (VIF) values below 5 (the acceptable threshold being 10), ruling out significant multicollinearity and ensuring model stability. Multivariate logistic regression was then applied to examine the influence of individual, organizational, community, and other multilevel factors on sufficient PA. The model exhibited excellent goodness of fit (Nagelkerke *R*^2^ = 0.992).

Table 6 showed that, at the individual level, self-efficacy was a key predictor of sufficient PA. Specifically, moderate (OR = 11.65, 95% CI: 2.52–53.79, *p* < 0.01) and high (OR = 39.45, 95% CI: 7.51–207.19, *p* < 0.001) self-efficacy levels were significantly positively associated with sufficient PA. At the interpersonal level, support from PE teachers 2–5 times per month significantly and positively predicted a sufficient level of PA (OR = 7.96, 95% CI: 2.10–30.25, *p* = 0.002).At the organizational level, sufficient school sports facilities were significantly associated with sufficient PA (OR = 5.15, 95% CI: 1.45–18.37, *p* = 0.011). At the community level, access to knowledge through multiple channels had notable predictor predictive effect on sufficient PA (OR = 11.31, 95% CI: 2.77–46.09, *p* = 0.001). No policy-level variables showed statistical significance (*p* > 0.05).

**Table 6:** Factors Associated with sufficient physical exercise between cases and controls through multiple logistic regression analysis (*n* = 222).

Independent variables		S.E	Wals	Sig.	OR	OR 的 95%C.I.	
		–	–	–	–	L	U
Individual Level							
Int. Motiv.	Low	–	–	–	1	–	–
	Medium	0.728	3.071	0.08	3.581	0.86	14.918
	High	0.743	1.568	0.21	2.536	0.591	10.877
Self-effic.	Low	–	–	–	1	–	–
	Medium	0.78	9.896	0.002	11.65	2.523	53.787
	High	0.846	18.862	0	39.453	7.513	207.19
Interpersonal Level Social Support							
Par. Supp. Knowl. (D1)	Cannot	–	–	–	1	–	–
	Can	0.723	3.337	0.068	3.746	0.908	15.452
Par. Emot. Supp. (D2)	Cannot	–	–	–	1	–	–
	Can	0.734	1.123	0.289	2.178	0.516	9.182
Par. Invest. (D3)	Cannot	–	–	–	1	–	–
	Can	0.733	0.419	0.517	1.607	0.382	6.753
Agree Views (D4)	Cannot	–	–	–	1	–	–
	Can	0.775	0.052	0.82	0.838	0.183	3.831
Par. Openness (D5)	Cannot	–	–	–	1	–	–
	Can	0.691	0.078	0.78	0.825	0.213	3.196
Par. Exer. Dur. (D7)	≤2 months	–	–	–	1	–	–
	3–4 months	0.699	1.646	0.199	0.408	0.104	1.605
	5–6 months	1.159	0.05	0.823	0.772	0.08	7.486
	≥6 months	1.31	0.294	0.588	2.034	0.156	26.52
PE Teacher Supp. (D9)	≤1 times/mos	–	–	–	1	–	–
	2–5 times/mos	0.681	9.282	0.002	7.963	2.096	30.252
	≥5 times/mos	0.759	2.65	0.104	3.443	0.777	15.255
Peer Encour. (D10)	≤1 times/mos	–	–	–	1	–	–
	2–5 times/mos	0.732	0.133	0.715	1.306	0.311	5.482
	≥5 times/mos	0.855	1.974	0.16	3.326	0.622	17.78
Peer Partic. (D11)	No	–	–	–	1	–	–
	Yes	0.642	0.487	0.485	1.565	0.445	5.505
Organizational Level							
Sch. Sports Fac. (E1)	No	–	–	–	1	–	–
	Yes	0.649	6.394	0.011	5.154	1.446	18.372
Extra Exer. Time (E2)	No	–	–	–	1	–	–
	Yes	0.602	0.163	0.687	0.784	0.241	2.552
Sch. Sports Comp. (E3)	No	–	–	–	1	–	–
	Yes	0.959	0.926	0.336	0.397	0.061	2.603
Sch. Track Meet (E4)	No	–	–	–	1	–	–
	Yes	0.969	0.919	0.338	2.533	0.379	16.93
Community Level							
Comm. Sports Fac. (F1)	No	–	–	–	1	–	–
	Yes	0.861	0.666	0.414	0.495	0.092	2.678
Comm. Sports Fac. Adeq. (F2)	No	–	–	–	1	–	–
	Yes	0.746	0.261	0.609	1.464	0.339	6.311
Comm. Sports Events (F3)	No	–	–	–	1	–	–
	Yes	0.788	3.394	0.065	4.266	0.911	19.97
Comm. Sports Partic. (F4)	No	–	–	–	1	–	–
	Yes	0.809	0.015	0.903	1.104	0.226	5.388
Free Train. (F5)	No	–	–	–	1	–	–
	Yes	0.727	1.469	0.226	2.415	0.58	10.05
Other Knowl. (F6)	No	–	–	–	1	–	–
	Yes	0.717	11.446	0.001	11.307	2.774	46.088
Policy Level							
Sch. PE Assess. (G1)	No	–	–	–	1	–	–
	Yes	0.865	0.58	0.446	0.518	0.095	2.82
Phys. Fit. Test (G2)	No	–	–	–	1	–	–
	Yes	0.844	0.817	0.366	2.144	0.41	11.199
intercept	–	1.515	34.629	0	0	–	–

## 4 Discussion

This matched case-control study involved 222 high school students from three high schools in Shanxi Province, China. Grounded in McLeroy's social ecological framework, we examined multilevel determinants of sufficient PA across five domains: individual, interpersonal, organizational, community, and policy. Key findings revealed that gender, grade level, economic status (monthly living expenses), self-efficacy, physical education teacher support, school sports facilities, and multi-channel PA knowledge acquisition were significant predictors of sufficient exercise engagement.

While no significant differences were found in household monthly income or parental occupation between groups, sufficient exercisers reported higher monthly living expenses. This suggests that immediate economic capacity for discretionary spending, rather than overall family socioeconomic status, may influence PA engagement in this urban sample. The homogeneity in household income within our urban school-based sample likely limited our ability to detect broader socioeconomic effects, indicating that in contexts with equitable educational resources, socioeconomic status may not be a primary determinant of PA behavior (30, 31). However, our findings suggest that in highly urbanized areas with equitable educational resources, household socioeconomic status may no longer be a decisive determinant of sufficient exercise behavior among high school students.

This study used multivariable binary logistic regression to identify determinants of sufficient PA adoption among students. After adjusting for confounders and mediators, several univariately significant factors became non-significant in the multivariate model. We retained only statistically significant predictors from the multivariate analysis. Consistent with previous literature, factors such as peer support and family support were excluded as they did not retain significance in multivariate adjustment (32, 33). The study identified self-efficacy in PA as a critical determinant of sufficient PA behavior among high school students. Sufficient exercisers exhibited significantly higher self-efficacy levels than insufficient exercisers, highlighting its pivotal role in sustaining PA engagement. This is consistent with established evidence (34), emphasizing self-efficacy's crucial role in both initiating and maintaining PA behaviors. Additionally, self-efficacy showed significant stage-specific variations across exercise behavior phases: it was lowest during the precontemplation stage, increased progressively through behavioral transitions, and peaked in the maintenance stage (35). These findings suggest that self-efficacy not only serves as a key predictor of exercise behavior but also potentially acts as a mediator through multiple mechanisms in the formation and maintenance of PA patterns (36). This further highlights its pivotal role as a core regulator bridging the intention-behavior gap in exercise adoption.

At the interpersonal level, support from physical education teachers played a key role in establishing sufficient PA patterns, consistent with Duncan et al.'s findings (37). However, some Western scholars suggest that parental and peer influences may have a greater impact than those of physical education or classroom teachers (38). This study challenges such propositions, likely reflecting China's unique sociocultural context, where the confucian tradition of reverence for educators, combined with high parental trust in educational authority and students' deep school immersion, strengthens institutional influence over familial or peer socialization (39). Unlike in many Western countries, Chinese physical educators(PE) are more directly involved in students' extracurricular activities, often providing exercise facilities or training alongside students—responsibilities typically handled by coaches or parents in other contexts (40).

At the organizational level, the adequacy of school sports facilities significantly predicted the establishment of sufficient exercise behaviors, consistent with existing evidence showing that institutional resources positively influence PA patterns (41). Crucially, providing quality sports facilities and diverse extracurricular activity opportunities enhances both the frequency and voluntary engagement of students in physical exercise. However, other studies have found limited direct predictive value of certain school factors—such as physical education class duration or facility utilization rates—on extracurricular PA engagement. As Hills et al. (42) note, while schools serve as critical venues for adolescent PA, curricular constraints often fail to provide sufficient activity, diminishing their immediate impact on extracurricular exercise behaviors. Overall, adequate provision of sports facilities in schools not only generates exercise opportunities but also actively encourages students' voluntary engagement and sustained participation in physical activities, as documented in our multilevel analysis.

At the community level, the dissemination of PA knowledge exerted a strong influence on students' sufficient exercise patterns. Serving as a key platform for transmitting PA information, the community significantly shapes the adoption and maintenance of exercise behaviors through various pathways (43). Research shows that the accessibility of community resources (e.g., facilities, training programs, and promotional initiatives) drives engagement in group PA behaviors (44). Furthermore, diverse knowledge acquisition pathways regarding physical exercise (e.g., media platforms and structured educational curricula) enhance public understanding of PA benefits, thereby increasing motivation and sustaining adherence to PA participation (45). This evidence reinforces the pivotal role of communities in fostering physically active cultures and shaping health behaviors.

Contrary to SEM-based expectations, policy-level factors were not significant independent predictors in this study. This may be due to the highly uniform implementation of national policies across urban schools, leaving limited variation to detect effects. Alternatively, policy influence may be indirectly channeled through downstream factors like organizational resources (sports facilities) or interpersonal support (teacher engagement) (46), which showed significance in our model.

In summary, the results extend social-ecological theory on adolescent PA and provide new evidence that self-efficacy, teacher support, and school- and community-level resources operate synergistically. Embedding teacher support within the Chinese cultural norm of “revere teachers, value education” can magnify its behavioural impact; integrating these assets at school and community levels is likely to yield meaningful gains in high-school students’ PA and subsequent health.

This study has several limitations. First, the sample was drawn from urban schools in one city, limiting generalizability to rural or other socioeconomic contexts. Second, the reliance on self-reported questionnaire data introduces potential social desirability bias, where participants may have overreported favorable PA behaviors. Objective monitoring via accelerometry was conducted for only 25% (*n* = 28) of the sufficiently active cohort, and no objective monitoring was performed for the control group. Additionally, PA level disparities among non-participating students were not reported. Although a case-control design is used, some variables (e.g., self-efficacy and PA behaviors) may have interactive effects, and large-scale studies should be conducted in the future.

## 5 Conclusion

Using a case-control design, this study systematically explored multilevel determinants of sufficient PA engagement among high school students. Key correlates included self-efficacy, support and instrumental assistance from PE teachers, the adequacy of school facilities, and diverse pathways for acquiring PA knowledge. In this study, these factors were significant predictors of adolescents' exercise engagement patterns. Therefore, in terms of implementation, it is essential to enhance students' self-efficacy, provide sustained teacher support and encouragement, ensure well-equipped school sports facilities, and facilitate convenient access to sports knowledge through multiple channels.

## Data Availability

The raw data supporting the conclusions of this article will be made available by the authors, without undue reservation.

## References

[1] Caspersen CJ Powell KE Christenson GM. Physical activity, exercise, and physical fitness: definitions and distinctions for health-related research. Public Health Rep. (1985) 100(2):126–31. 3920711 PMC1424733

[2] Bull FC Al-Ansari SS Biddle S Borodulin K Buman MP Cardon G World health organization 2020 guidelines on physical activity and sedentary behaviour. Br J Sports Med. (2020) 54(24):1451–62. 10.1136/bjsports-2020-102955 33239350 PMC7719906

[3] Strain T Flaxman S Guthold R Semenova E Cowan M Riley LM National, regional, and global trends in insufficient physical activity among adults from 2000 to 2022: a pooled analysis of 507 population-based surveys with 5·7 million participants. Lancet Glob Health. (2024) 12(8):e1232–43. 10.1016/S2214-109X(24)00150-5 38942042 PMC11254784

[4] Rakić JH Dzielska Z Felder-Puig A Oja R Bakalár L Nardone P A focus on adolescent physical activity, eating behaviours, weight status and body image in Europe, Central Asia and Canada. Health Behaviour in School-aged Children International Report from the 2021/2022 Survey. Vol. 41–9. (2024). Available online at: https://hbsc.org/publications/reports/a-focus-on-adolescent-physical-activityeating-behaviours-weight-status-and-body-image-in-europe-central-asia-and-canada/ (Accessed December 12, 2024).

[5] Farooq MA Parkinson KN Adamson AJ Pearce MS Reilly JK Hughes AR Timing of the decline in physical activity in childhood and adolescence: gateshead millennium cohort study. Br J Sports Med. (2018) 52(15):1002–6. 10.1136/bjsports-2016-096933 28288966 PMC6204977

[6] Wu Y Qin G Wang G Liu L Chen B Guan Q Physical activity, sedentary behavior, and the risk of cardiovascular disease in type 2 diabetes mellitus patients: the MIDiab study. Engineering. (2023) 20:26–35. 10.1016/j.eng.2022.05.013

[7] Abarca-Gómez L Abdeen ZA Hamid ZA Abu-Rmeileh NM Acosta-Cazares B Acuin C Worldwide trends in body-mass index, underweight, overweight, and obesity from 1975 to 2016: a pooled analysis of 2416 population-based measurement studies in 128·9 million children, adolescents, and adults. Lancet. (2017) 390(10113):2627–42. 10.1016/S0140-6736(17)32129-3 29029897 PMC5735219

[8] Mathisen FKS Torsheim T Falco C Wold B. Leisure-time physical activity trajectories from adolescence to adulthood in relation to several activity domains: a 27-year longitudinal study. Int J Behav Nutr Phys Act. (2023) 20(1):27. 10.1186/s12966-023-01430-4 36890586 PMC9996998

[9] Liu G Li W Li X. Striking a balance: how long physical activity is ideal for academic success? Based on cognitive and physical fitness mediation analysis. Front Psychol. (2023) 14:1226007. 10.3389/fpsyg.2023.1226007 37519383 PMC10372443

[10] Nagata JM Vittinghoff E Pettee Gabriel K Garber AK Moran AE Rana JS Moderate-to-vigorous intensity physical activity from young adulthood to middle age and metabolic disease: a 30-year population-based cohort study. Br J Sports Med. (2022) 56(15):847–53. 10.1136/bjsports-2021-104231 34521685 PMC9017156

[11] Sayyah M Etemad Hosseini M Dibahosseini SA. Comparing the physical fitness conditions of boys’ school students in the city of Kashan. AI Tech Behav Soc Sci. (2024) 2(2):7–11. 10.61838/kman.aitech.2.2.2

[12] Hearst MO Patnode CD Sirard JR Farbakhsh K Lytle LA. Multilevel predictors of adolescent physical activity: a longitudinal analysis. Int J Behav Nutr Phys Act. (2012) 9(1):8. 10.1186/1479-5868-9-8 22309949 PMC3305547

[13] McLeroy KR Bibeau D Steckler A Glanz K. An ecological perspective on health promotion programs. Health Educ Q. (1988) 15(4):351–77. 10.1177/109019818801500401 3068205

[14] Kiyani T Kayani S Kayani S Batool I Qi S Biasutti M. Individual, interpersonal, and organizational factors affecting physical activity of school adolescents in Pakistan. Int J Environ Res Public Health. (2021) 18(13):7011. 10.3390/ijerph18137011 34209078 PMC8296940

[15] Hu D Zhou S Crowley-McHattan ZJ Liu Z. Factors that influence participation in physical activity in school-aged children and adolescents: a systematic review from the social ecological model perspective. Int J Environ Res Public Health. (2021) 18(6):3147. 10.3390/ijerph18063147 33803733 PMC8003258

[16] Xie C Zhang Z Zhang X Li Y Shi P Wang S. Effects of interventions on physical activity behavior change in children and adolescents based on a trans-theoretical model: a systematic review. BMC Public Health. (2025) 25(1):657. 10.1186/s12889-025-21336-z 39966763 PMC11834675

[17] Keyes CR. Parent-teacher partnership: a theoretical approach for teachers. In: Katz L, editor. Issues in Early Childhood Education: Curriculum, Teacher Education & Dissemination of Information, Proceedings of the Lilian Katz Symposium; 2000 Nov 5–7; Champaign, IL, USA. Champaign, IL: University of Illinois at Urbana-Champaign (2000). p. 107–118.

[18] Srivastav P Vaishali K Rajwar E Broadbent S Bhat HV. Factors associated with physical activity participation among children: a systematic review protocol. Syst Rev. (2023) 12(1):70. 10.1186/s13643-023-02226-0 37106415 PMC10134558

[19] Craig CL Marshall AL Sjöström MM Bauman AE Booth ML Ainsworth BE International physical activity questionnaire: 12-country reliability and validity. Med Sci Sports Exerc. (2003) 35(8):1381–95. 10.1249/01.MSS.0000078924.61453.FB 12900694

[20] Rääsk T Mäestu J Lätt E Jürimäe J Jürimäe T Vainik U Comparison of IPAQ-SF and two other physical activity questionnaires with accelerometer in adolescent boys. PLoS One. (2017) 12(1):e0169527. 10.1371/journal.pone.0169527 28056080 PMC5215940

[21] Cain KL Sallis JF Conway TL Van Dyck D Calhoon L. Using accelerometers in youth physical activity studies: a review of methods. J Phys Act Health. (2013) 10(3):437–50. 10.1123/jpah.10.3.437 23620392 PMC6331211

[22] Wang X Ji X. Sample size estimation in clinical research. Chest. (2020) 158(1):S12–20. 10.1016/j.chest.2020.03.010 32658647

[23] Likert R. A technique for the measurement of attitudes. Arch Psychol. (1932) 22(140):55–55.

[24] Guay F Vallerand RJ Blanchard C. On the assessment of situational intrinsic and extrinsic motivation: the situational motivation scale (SIMS). Motiv Emot. (2000) 24(3):175–213. 10.1023/A:1005614228250

[25] Ying J. Development of the physical activity self-efficacy scale for college students. Adv Psychol. (2018) 8(12):1769–77. 10.12677/AP.2018.812206

[26] Brown TA. Confirmatory Factor Analysis for Applied Research. New York: Guilford Press (2006). p. 475. (Methodology in the social sciences).

[27] Field A. Discovering statistics using bm spss statistics. 5th ed. London: Sage Publications (2024). p. 371.

[28] Hosmer DW Lemeshow S. Applied Logistic Regression. 2nd ed. New York: Wiley (2000). p. 373. (Wiley series in probability and statistics).

[29] Freedson PS Melanson E. Calibration of the computer science and applications, Inc. accelerometer. Med Sci Sports Exerc. (1998) 30(5):777–81. 10.1097/00005768-199805000-00021 9588623

[30] Gautam N Dessie G Rahman MM Khanam R. Socioeconomic status and health behavior in children and adolescents: a systematic literature review. Front Public Health. (2023) 11:1228632. 10.3389/fpubh.2023.1228632 37915814 PMC10616829

[31] Wolfe AM Lee JA Laurson KR. Socioeconomic status and physical fitness in youth: findings from the NHANES national youth fitness survey. J Sports Sci. (2020) 38(5):534–41. 10.1080/02640414.2020.1713688 31952463

[32] Villar E Real-Deus E Martínez-López Z Mayo ME Tinajero C. Perceived peer support, motivational self-regulation and academic achievement in adolescents. Br J Educ Psychol. (2025):bjep.12783. 10.1111/bjep.12783 PMC12590935 40388556

[33] Hohepa M Scragg R Schofield G Kolt GS Schaaf D. Social support for youth physical activity: importance of siblings, parents, friends and school support across a segmented school day. Int J Behav Nutr Phys Act. (2007) 4(1):54. 10.1186/1479-5868-4-54 17996070 PMC2186357

[34] Chen Y Yao SJ Ma QS Shao W Liu C Guo KL. The relationship between exercise intention and exercise behavior of junior school students: an analysis of chain mediating effect. Front Psychol. (2022) 13:935264. 10.3389/fpsyg.2022.935264 36003092 PMC9394673

[35] Oka K. [Stages of change for exercise behavior and self-efficacy for exercise among middle-aged adults]. Nihon Koshu Eisei Zasshi Jpn J Public Health. (2003) 50(3):208–15. 12704833

[36] Parschau L Fleig L Warner LM Pomp S Barz M Knoll N Positive exercise experience facilitates behavior change via self-efficacy. Health Educ Behav. (2014) 41(4):414–22. 10.1177/1090198114529132 24722218

[37] Duncan SC Duncan TE Strycker LA. Sources and types of social support in youth physical activity. Health Psychol. (2005) 24(1):3–10. 10.1037/0278-6133.24.1.3 15631557

[38] Hagger M Chatzisarantis NLD Hein V Soós I Karsai I Lintunen T Teacher, peer and parent autonomy support in physical education and leisure-time physical activity: a trans-contextual model of motivation in four nations. Psychol Health. (2009) 24(6):689–711. 10.1080/08870440801956192 20205021

[39] Sang K. A comparative study of differences between Chinese and American family educational approaches. J Educ Theory Manag. (2017) 1(1):70. 10.26549/jetm.v1i1.295

[40] Konukman F Agbuĝa B Erdoĝan Ş Zorba E Demirhan G Yilmaz I. Teacher-coach role conflict in school-based physical education in USA: a literature review and suggestions for the future. Biomed Hum Kinet. (2010) 2:19–24. 10.2478/v10101-010-0005-y

[41] Wang L Tang Y Luo J. School and community physical activity characteristics and moderate-to-vigorous physical activity among Chinese school-aged children: a multilevel path model analysis. J Sport Health Sci. (2017) 6(4):416–22. 10.1016/j.jshs.2017.09.001 30356647 PMC6189256

[42] Hills AP Dengel DR Lubans DR. Supporting public health priorities: recommendations for physical education and physical activity promotion in schools. Prog Cardiovasc Dis. (2015) 57(4):368–74. 10.1016/j.pcad.2014.09.010 25269062

[43] Guo X Yang X Mao S. Study on the impact of rural public sports facilities and instructors on residents’ participation in sports activities in China. Front Public Health. (2025) 13:1475321. 10.3389/fpubh.2025.1475321 40078774 PMC11897029

[44] Martínez C. The role of community sports programs in promoting physical activity among adolescents in Belgium. Rev Psicol Deporte J Sport Psychol. (2024) 33(2):186–95.

[45] Den Braver NR Garcia Bengoechea E Messing S Kelly L Schoonmade LJ Volf K The impact of mass-media campaigns on physical activity: a review of reviews through a policy lens. Eur J Public Health. (2022) 32(Supplement_4):iv71–83. 10.1093/eurpub/ckac085 36444108 PMC9706123

[46] O’Toole LJ Montjoy RS. Interorganizational policy implementation: a theoretical perspective. Public Adm Rev. (1984) 44(6):491. 10.2307/3110411

